# Evolution of Volatile Flavour Compounds during Fermentation of African Oil Bean (*Pentaclethra macrophylla* Benth) Seeds for “Ugba” Production

**DOI:** 10.1155/2015/706328

**Published:** 2015-01-20

**Authors:** C. O. Nwokeleme, J. Obeta Ugwuanyi

**Affiliations:** Department of Microbiology, University of Nigeria, Nsukka 410001, Nigeria

## Abstract

Fermented African oil bean (*Pentaclethra macrophylla* Benth) seed is a successful and well studied seasoning and snack in parts of Western Africa. GC-MS analysis of fermenting seeds revealed a mixture of several volatile aroma compounds which changed with time and starter organism. During natural mixed culture process 36 volatile compounds including 12 hydrocarbons, 10 esters, 5 alcohols, 2 phenols, 2 ketones, and one each of furan, amine, acid, thiophene, and lactone were identified. When *Bacillus subtilis* was used in pure culture, 30 compounds comprising 10 hydrocarbons, 8 esters, 3 alcohols, 2 amines, 2 sulfur compounds, and one of each of acid, aldehyde, phenol, ketone, and furan were identified. Sample fermented with *B. megaterium* produced 29 aroma compounds comprising 9 hydrocarbons, 10 esters, 2 nitrogenous compounds, 2 ketones, 3 alcohols, and one of each of lactone, aldehyde, furan, and amine. Methyl esters of various long chain fatty acids may be key aroma compounds, based on consistency and persistence. Qualitative or quantitative contribution of individual compounds may only be determined following flavour threshold analysis.

## 1. Introduction

African oil bean (*Pentaclethra macrophylla* Benth) is a member of the family Leguminosae. It is popular in Nigeria where it is known by several names such as* Apara* in Yoruba,* Ukana* in Efik, and, the most prominent,* Ugba/Ukpaka* in Igbo.* Ugba* is a popular Igbo condiment and delicacy made from traditional household solid state fermentation of African oil bean seeds. The fermentation is a mixed culture alkaline process involving a variety of microorganisms [1, 2]. Although a variety of microorganisms are involved in the process, only the* Bacillus *spp. appear to be necessary for the development of typical flavour [2–6].

The methods of production and also the length of fermentation vary from one producer to another and with the final intended use resulting in nonuniform products. However, the basic method involves boiling the seeds for up to 12 hours, removing and slicing the cotyledons, soaking/washing the sliced cotyledons in several changes of water, and wrapping the sliced cotyledons for the fermentation to take place [1]. Beans that have been fermented for 2-3 days are taken as a snack delicacy directly or following seasoning and some further processing. Well fermented beans (up to 5 or more days) are added to soup as flavouring. Prepared in different ways, Ugba is an essential food item for various traditional ceremonies, and in instances it may be used as meat substitute in certain soups/gravies particularly for the rural poor [1].

Flavour is considered the quality index of* Ugba* [7] and plays an important role in consumer acceptability. Although fermented African oil bean seeds have typical flavour and appealing organoleptic quality, differences in flavour range and intensities exist. These vary perhaps as attributes of producer organisms and are due to the various volatile compounds produced by the fermenting population [8]. A great deal of work has been implemented in the microbiological characterisation of the fermentation process. However, unlike Asian and Pacific fermented seasoning, no work has yet been reported, to our knowledge, on the flavour components of Ugba and their evolution during the fermentation process. In fact, this knowledge gap is not restricted to Ugba but appears to cut across the entire gamut of African fermented foods, particularly of the genre of seasoning agents.

Knowledge of the volatile and flavour characteristics of the fermented oil bean seeds and their evolution will aid in the quality control of the process, modification of the flavour, and eventual synthesis of the Ugba flavour products for possible use in the seasoning of various factory processed and even home foods. This is important in view of declining production of seeds, due to deforestation, and the need to extend use of the appealing aroma of Ugba to other food types. The aim of this study was to identify the volatile flavour components of African oil bean seeds during natural and pure culture fermentation and to relate these to the fermentation progress.

## 2. Materials and Method

### 2.1. Traditional Production of Ugba

African oil bean seeds were purchased from dealers in a local market in Nsukka, Enugu State. Ugba was prepared in the laboratory using the traditional method [1]. The seeds were first boiled for 6–8 hrs to soften the coat and enhance peeling. The cotyledons were then sliced longitudinally into 4.5 cm × 0.1-0.2 cm slices, washed and boiled in water for 1-2 hrs, and drained and soaked in water for 10–12 hrs. After soaking, the slices were washed in several changes of water to reduce bitterness and allowed to drain in a basket. The slices were then wrapped in clean dry banana leaves (*Musa sapientum* Linn.) in 40–50 g wraps. The wraps were allowed to ferment for 3 days at room temperature (28 ± 2°C) to produce Ugba. Traditional fermentation was implemented in triplicate.

### 2.2. Pure Culture Production of Ugba

*Ugba* was produced in the laboratory according to the method described by Obeta [1]. One kilogram (1 kg) of African oil bean seeds was boiled dehulled, peeled, washed, and sliced for the traditional process. The slices were further washed and 20 g portions were placed into conical flasks and capped tightly with cotton wool and aluminium foil. The flasks containing the slices were sterilized by autoclaving at 110°C for 10 mins. Triplicate flasks were set up for pure culture process.

### 2.3. Development of Pure Isolates as Starters

Pure cultures of* B. subtilis *and* B. megaterium* isolated from the traditional process and identified by standard microbiological procedures were inoculated independently in 20 mL sterile 0.1% peptone water and incubated for 24 hrs. After incubation, the cultures were centrifuged and the culture pellets were washed repeatedly with sterile saline and distilled water. 0.1 mL of the cells were emulsified in 9.9 mL distilled water and pour plate method was used to ascertain the population of the cells and to check for culture purity.

### 2.4. Pure Culture Fermentation

Each flask was separately, aseptically inoculated with 1 mL suspension (5.0 × 10^9^ mL^−1^) of* B. subtilis *or* B. megaterium* individually using a sterile pipette and mixed with the sample by simple manual mixing. Fermentation was allowed to proceed for 72 hrs at room temperature with occasional manual mixing by shaking the flask. To check for purity of the process during the period of fermentation, 1 g of the fermenting slices was removed at 24-hour interval and at the end of fermentation and agitated in 9 mL 0.1% sterile peptone water. Smear of the sample was streaked on nutrient agar plate for isolation of the particular organism acting as a single pure culture. Triplicate pure culture flasks were homogenised and sampled for analysis of volatile compounds. Only pure culture processes were analysed for volatile compounds.

### 2.5. Analysis for Volatile Flavour Compounds: Sampling and Extraction Procedure

Samples for analysis were collected from naturally fermented Ugba and also from Ugba fermented with pure cultures of* B. subtilis* and* B. megaterium*. Samples were collected at 12-hour intervals for 3 days. One gram (1 g) of the fermenting sample was ground and homogenized in 9 mL diethyl ether to extract volatile flavour compounds. Whatman No. 1 filter paper was used to separate the mixture; then Millipore Filtration using membrane filter of 0.45 *μ*m pore size was used to remove particulate matter from the resulting filtrate. The resulting filtrate was placed in small ampoules, sealed, and stored at −30°C until being required for analysis.

### 2.6. GC-MS Analysis

Analyses were done on GC-MS (QP 2010 plus; Shimadzu Corp, Japan) operated in electron-impact (EI) mode with an ionization voltage of 70 eV. Sample aliquot of 1 *μ*L was injected and analyses were done with split injection mode. The GC column oven temperature was 60°C and injector temperature was 250°C with 53.2 mL/min flow and interface temperature of 250°C. Helium at 56.2 kPa was employed as the carrier gas. Oven temperature was held at 60°C for 2 mins, ramped to 180°C and held for 3 mins, and finally programmed at 280°C and held for 5 mins. The ion source temperature was 200°C and the solvent cut time was 2.50 mins. GC chromatograms of the fermenting African oil bean seeds were examined and each peak identified was confirmed by direct comparison of their chromatographic retention times, retention indices, and mass spectra with those of the authentic compounds and/or data in the NIST05 Mass Spectral Library.

## 3. Results

### 3.1. Traditional Production of Ugba

At the end of the traditional fermentation, the produced Ugba had a very light brown colour and exhibited classical aroma of the product, with a light, but sharp, ammoniacal smell. There was no visible sign of any slime on the slices which remained firm but slightly softer than the control/starting material. The product tasted like the traditional market samples with a slight distant bitter tinge. A total of 36 aroma compounds made up of 12 hydrocarbons, 10 esters, 5 alcohols, 2 phenols, 2 ketones, and one of each of furan, amine, acid, thiophene, and lactone were identified at different fermentation periods during natural fermentation (Table 1).

### 3.2. Pure Culture Production of Ugba

At the end of the pure culture process, the products of fermentation with two organisms were generally similar in colour, both being very light brown with no visible slime on the slices. The product of the* B. subtilis *fermentation, however, had only a slightly deeper ammoniacal smell than that of* B. megaterium. *Both products tasted similar to the product of natural process but were slightly bitterer than the natural process. Similarly, the product of* B. megaterium *was bitterer than that made with* B. subtilis. *The extract of oil seed fermented with* B. subtilis* yielded 30 aroma compounds comprising 10 hydrocarbons, 8 esters, 3 alcohols, 2 sulfur compounds, 2 amines, and one of each of acid, aldehyde, phenol, ketone, and furan (Table 2). On the other hand, sample fermented with* B. megaterium* produced 29 aroma compounds consisting of 9 hydrocarbons, 10 esters, 2 nitrogenous compounds, 2 ketones, 3 alcohols, and one of each of lactone, aldehyde, furan, and amine (Table 3).

## 4. Discussion

Volatile compounds have been shown to be partly responsible for the aroma of several fermented foods [7–11]. They are probably important players in the flavour of Ugba, as they have been shown to be important in a variety of* Bacillus *spp. fermented foods [2]. Ugba flavours are formed during fermentation as a result of breakdown of plant compounds as well as formation of microbial metabolites by the active populations responsible for the fermentation process. The ammoniacal flavour observed in Ugba is believed to result from the alkaline degradation of protein component of the seed by the fermenting* Bacillus *spp. [7]. Many of the aroma compounds identified in this study derived from the breakdown of lipids (fatty acid), proteins (amino acid), and other bioavailable compounds in Ugba through the activities of the microbial enzymes. Hydrocarbons, esters, alcohols, phenols, ketones, furan, and amines were detected in all the samples examined and the consistency of these various volatiles would suggest roles for them in the final flavour of the product, although minor. However, neither the flavour thresholds of these compounds in this product nor their precise individual contributions are yet known.

Hydrocarbons are assumed to be formed from fatty acid by lipoxygenase catalysis [12]. This may explain their abundance as volatile compounds in the fermented African oil bean seeds, since the seeds are rich in oil [13]. Nakamura et al. [14] suggested that hydrocarbons do not play a significant role as flavour compounds in roasted sesame since they possess a relatively weak aroma. If this be the case in Ugba, then the principal flavour compounds may be other compounds coproduced in the fermentation process. It is to be expected, as with all complex products, that the final flavour of Ugba will derive from the interaction of a variety of compounds.

Methyl esters of fatty acids are one of the largest group of volatile aroma compounds produced during the fermentation in both mixed culture natural fermentation and pure culture processes. These were largely produced during 36–48 hr of fermentation and persisted in various forms to the end of the process. They are produced enzymatically during fermentation by the methanolysis of acyl-COA that is formed during fatty acid synthesis or degradation. These compounds have been reported to be responsible for quality sensory properties of various fermented foods [15]. 9-Octadecenoic acid, methyl ester; pentanoic acid, methyl ester; 11,14-eicosadienoic acid, methyl ester; and methyl 12-methyl tetradecanoate are methyl esters of fatty acids which occurred commonly and in significant amounts (from the peak areas) in all the samples.

Unfermented (0 hr) samples contained 2-butyltetrahydrofuran which rapidly disappeared as fermentation progressed. Furanic compounds are among the aroma compounds obtained from carbohydrate degradation [16]. This is consistent with the report of high carbohydrate content of unfermented* Pentaclethra macrophylla *[17]. Histamine was identified at 0 hr fermentation and cyclopentanamine was identified in the sample fermented with* B. subtilis* at 36 hr. The presence of these compounds may be attributed to the enzymatic decarboxylation of amino acid in the seeds during fermentation. The presence of nitrogenous compounds, butyl isocyanide and acetonitrile, in the sample fermented with* B. megaterium* at 12 hr and 48 hr, is indicative of deaminase activity during the fermentation. Unfermented Ugba has been reported to contain cyanide and other toxic compounds/antimetabolites [18] as has been reported in a variety of oil seeds [19]. Some volatile sulfur compounds in food are obtained by a variety of enzymatic reactions and/or as products of secondary metabolism of leucine and cysteine [12]. Methane sulfonic anhydride and t-butyl sulfinate, O-methyl, were present at 12 hr and 60 hr, respectively, in the sample fermented with* B. subtilis*.

Considerable amounts of various alcohols were identified during the fermentation in all samples. Of these, 1,14-tetradecanediol and 1,10-decanediol occurred severally at different periods of fermentation in all the samples. 3,7-Dimethyl-7-octen-1-ol was identified in the sample fermented with* B. megaterium.* Many alcohols have been described as possessing undesirable odor which partially contributes to “raw” or “beany” flavour of soya bean [20]. A small number of aldehydes, ketones, and acids were identified in this study. Fatty acids and amino acids are precursor of a great number of volatile aldehydes [12]. It is conceivable that these compounds contribute to the final flavour of fermented African oil bean seeds. Aldehyde can also be obtained as metabolic by-products of the enzymatic transamination or oxidative deamination of amino acid. 2,4-Hexadienal and 14-heptadecenal are aldehydes identified in the samples fermented with* B. subtilis* and* B. megaterium* at 60 hr of fermentation. However, they did not persist nor were they present in the natural process. Although* B. subtilis* and* B. megaterium* are present in natural fermentation process the profile of volatiles differed between pure culture and traditional one. This is understandable, as the mixture of other microorganisms present in the natural process may modulate the variety, quantity, and persistence of different volatile compounds.

Phenolic compounds when present in fermented foods impart smoky and/brown odour [14]. Phenol, 2,4-bis(dimethylbenzyl) phenol, and 2,4-bis(dimethylbenzyl)-6-t-butylphenol identified during natural fermentation were not detected in pure culture fermentation and may be attributed to the smoked banana leaves used in wrapping the slices prior to fermentation. Although relatively small amounts of aldehyde, ketone, furans, amines, lactones acids, and thiophenes were identified in this study, they are expected to play important roles in the aroma of Ugba because of their low detection/aroma thresholds as has been reported in roasted chicory [21], in the dry scented Thai flowers used in tea production [22] and in roasted Malaysian almond nuts [23].

## 5. Conclusion

A vast array of volatile compounds has been identified in fermented* Pentaclethra macrophylla* seeds (Ugba). The variety and quantity of these volatiles change with the time of fermentation and the participating populations. The dynamic nature of evolution of volatile compounds is interesting for modulating the flavour of the final product. It remains to be demonstrated, the relative amounts, flavour thresholds, and specific contribution of the various compounds to the final flavour of the ready to eat fermented seeds. Clearly, a proper understanding of the roles of the various flavour compounds will assist in the modulation of Ugba flavour for the various possible uses of this important fermented seasoning and for a possible use in food processing industries and homes as a seasoning.

## Figures and Tables

**Table 1:** Volatile compounds identified during natural fermentation of African oil bean seed.

Fermentation time (hrs)/identified flavour compounds						
0	12	24	36	48	60	72
2,4,6-Tritert-butyl-4-methyl-2,5-cyclohexadien-1-one. 2-Butyl tetrahydrofuran. (2-Cyclohexylidene ethyl) cyclohexane. Pentanoic acid, 10-undecenyl ester. Histamine.	(5-Ethylcyclopent-1-enyl) methanol. 1-Hexyl-2-nitrocyclohexane. 2,4-Bis(dimethylbenzyl)phenol. 2,4-Bis(dimethylbenzyl)-6-t-butyl phenol.	S-[3-(2-Methyl[1,3]dioxolan-2-yl)-2-oxopropyl]ester. 1-Hexyl-1-nitrocyclohexane. 2-Methylheptanoic acid. Cyclohexapropanol. 1,14-Tetradecanediol. 3-Methyl-2-tridecylthiophene.	Tridecanoic acid, methyl ester. 11,14-Eicosadienoic acid, methyl ester. 9-Octadecenoic acid, methyl ester. Tetradecanoic acid, methyl ester. 1-Iodononane. 2,3,3-Trimethyloctane. 2,6,10,14-Tetramethylheptadecane. 3-Cyclohexyl-1-propanol 1,10-decanediol. 3,8-Dimethylundecane. Nonadecane. 7-Hexylicosane.	Z-4-Dodecenol 5,6-Undecadiene. (Z) 6-Pentadecen-1-ol.	Oxalic acid, dineopentyl ester. 1-Iodononane. Isobutyric acid, allyl ester. Butanoic acid, 2-butoxy-1-methyl-2-oxoethyl ester. Cyclopropyl methyl ketone. (Z)-Cyclododecene. 1,14-Tetradecanediol.	2,4,6-Tritert-butyl-4-methyl-2,5-cyclohexadien-1-one. Methyl 12-methyltetradecanoate. 11,14-Eicosadienoic acid, methyl ester. 9-Octadecenoic acid (Z), methyl ester. Methyl isoheptadecanoate. Isobutylcyclohexane 5,7-Dimethyl-1,6-octadiene. (Z)6,(Z)9-Pentadecadien-1-ol. 2,4-Bis(dimethylbenzyl)-6-t-butylphenol.

**Table 2:** Volatile compounds identified during pure culture fermentation of African oil Bean seed with *B. subtilis*.

Fermentation time (hrs)/identified flavour compounds						
0	12	24	36	48	60	72
2,4,6-Tritert-butyl-4-methyl-2,5-cyclohexadien-1-one. 2-Butyl tetrahydrofuran. (2-Cyclohexylidene ethyl)cyclohexane. Pentanoic acid, 10-undecenyl ester. Histamine.	2-Methylpentanoic acid. Sorbic acid vinyl ester. 9-Octadecenoic acid (Z), methyl ester. Methyl 12-methyl tetradecanoate. Phosphorous acid, tris(2-ethylhexyl) ester. Methanesulfonic anhydride.	Oxalic acid, cyclobutyl nonyl ester. 1-Iodoundecane. Hexadecane. Nonadecane. 9-Decen-1-yl acetate 1,14-Tetradecanediol Sulfurous acid, 2-ethyl hexyl isohexyl ester. Tetratetracontane. Eicosane.	Phosphonous dichloride, chloro(1,1-dimethylethyl) phosphinomethyl. Cyclopentanementhol, 3-methylene. 1,1,3-Trimethyl cyclopentane. Cyclopentanamine.	4,4-Dimethyl-1-hexene. Cyclopenta[d]anthracene-8,11-diol,3-isopropyl- 1,2,3,3a,4,5,6,6a,7,12-decahydro-,dibenzoate.	2,4-Hexadienal,(E,E). t-butylsulfinate,-O-menthyl. (Z)-Cyclododecene. 1,14-Tetradecanediol. 9-Octadecenoic acid (Z), 2,3-dihydroxypropyl ester.	Phosphonous dichloride, chloro (1,1-dimethylethyl) phosphinomethyl. Hexylidencyclohexane. Pentanoic acid, 10-undecenyl ester. 2,6-Ditert-butyl-4-(2,3,4,5,6-pentafluorobenzyl) phenol.

**Table 3:** Volatile compounds identified during pure culture fermentation of African oil bean seed with *B. megaterium*.

Fermentation time (hrs)/identified flavour compounds						
0	12	24	36	48	60	72
2,4,6-Tritert-butyl-4-methyl-2,5-cyclohexadien-1-one. 2-Butyltetrahydrofuran. (2-Cyclohexylideneethyl) cyclohexane. Pentanoic acid, 10-undecenyl ester. Histamine.	Butyl isocyanide. Acetonitrile. 2-Propyn-1-ol.	Methyl 14-methylpentadecanoate. 11,14-Eicosadienoic acid, methyl ester. 9-Octadecenoic acid (Z), methyl ester. Methyl isoheptadecanoate. 11,14,17-Eicosatrienoic acid, methyl ester. Oxalic acid, dineopentyl ester. 3-Ethyl-3-methylheptane. Sulfurous acid, hexyl octyl ester. 3-ethyl-4,5-dimethyl-1,4-Hexadiene. tetradecahydro-Benzocyclodecene. 2,4,4-Trimethylhexane. Pentanoic acid, methyl ester. 2,3,8-Trimethyldecane. 4-Methylnonane.	Tridecanoic acid, methyl ester. 11,14-Eicosadienoic acid, methyl ester. 9-Octadecenoic acid, methyl ester. Methyl isoheptadecanoate. 3,7-Dimethyl-7-octen-1-ol.	Butyl isocyanide. 1H-1,2,3-Triazole. 6-tert-Butyl-2,8a-dimethyloctahydro-1 (2H)-napthalenone. 3,7-Dimethyl-7-octen-1-ol.	6-(1-hydroxy-3-methylbutyl)-7-methoxy-2H-1-Benzopyran-2-one. 9-Octadecenoic acid (Z)-, methyl ester. 14-Hepta decanal.	2,4,6-Tritert-butyl-4-methyl-2,5-cyclohexadien-1-one. (4E)-2,3,3-Trimethyl-4-nonene. (Z)-Cyclododecene. 1,10-Decanediol.
